# Congenital rubella syndrome and autism spectrum disorder prevented by rubella vaccination - United States, 2001-2010

**DOI:** 10.1186/1471-2458-11-340

**Published:** 2011-05-19

**Authors:** Brynn E Berger, Ann Marie Navar-Boggan, Saad B Omer

**Affiliations:** 1 Department of Epidemiology, Rollins School of Public Health, Emory University, 1518 Clifton Road NE, Room 7017 (CNR Building), Atlanta, Georgia 30322, USA; 2 Departments of Pediatrics and Internal Medicine, Duke University Medical Center, Durham, North Carolina, USA; 3 Hubert Department of Global Health, Rollins School of Public Health, Emory University, Atlanta, Georgia, USA; 4 Department of Pediatrics, Emory University School of Medicine, Atlanta, Georgia USA; 5 Emory Vaccine Center, Atlanta, Georgia, USA

## Abstract

**Background:**

Congenital rubella syndrome (CRS) is associated with several negative outcomes, including autism spectrum disorders (ASDs). The objective of this study was to estimate the numbers of CRS and ASD cases prevented by rubella vaccination in the United States from 2001 through 2010.

**Methods:**

Prevention estimates were calculated through simple mathematical modeling, with values of model parameters determined from published literature. Model parameters included pre-vaccine era CRS incidence, vaccine era CRS incidence, the number of live births per year, and the percentage of CRS cases presenting with an ASD.

**Results:**

Based on our estimates, 16,600 CRS cases (range: 8300-62,250) were prevented by rubella vaccination from 2001 through 2010 in the United States. An estimated 1228 ASD cases were prevented by rubella vaccination in the United States during this time period. Simulating a slight expansion in ASD diagnostic criteria in recent decades, we estimate that a minimum of 830 ASD cases and a maximum of 6225 ASD cases were prevented.

**Conclusions:**

We estimate that rubella vaccination prevented substantial numbers of CRS and ASD cases in the United States from 2001 through 2010. These findings provide additional incentive to maintain high measles-mumps-rubella (MMR) vaccination coverage.

## Background

Rubella is a significant public health concern, as maternal rubella infection during pregnancy can lead to congenital rubella syndrome (CRS) in the fetus [1]. CRS comprises various defects, including deafness, cataracts, encephalitis, heart abnormalities, and mental retardation, among others [1,2]. The severity of CRS depends on the time of infection during gestation, with the most serious complications resulting from maternal infection in the first trimester [2]. The largest rubella epidemic occurred in the United States in the mid-1960s, when more than 20,000 children were born with CRS after an outbreak of over 12.5 million cases of rubella during 1963-1965 [3-5]. Prenatal rubella infection also led to thousands of fetal and infant deaths [6].

After the epidemic, several large-scale studies were conducted on the so-called "rubella children," establishing a firm link between prenatal rubella infection and congenital disorders [1,3,4]. Moreover, Chess found that autism is one of the many outcomes associated with CRS [7].

Using simple mathematical modeling, we calculated the number of CRS and ASD cases that were prevented by rubella vaccination in the United States from 2001 through 2010. We also performed sensitivity analyses to examine how changes in certain model parameters affect these prevention estimates.

## Methods

In our simple model, the number of CRS cases prevented by rubella vaccination in the United States during the ten-year period from 2001 through 2010 (*X *) is given by(1)

and the corresponding number of prevented ASD cases (*Y *) is given by(2)

where *α *and *β *are vaccine era and pre-vaccine era CRS incidence in the US, respectively, and *γ *is average number of live births per year. The percentage of CRS cases presenting with an ASD is denoted by *δ *. This model was chosen as a parsimonious representation of the relationship between a reduction in CRS incidence resulting from rubella vaccination and the incidence of ASD.

Table 1 defines the parameters used to calculate the numbers of CRS and ASD cases prevented by rubella vaccination in the United States from 2001 through 2010. The estimate of vaccine era CRS annual incidence (*α *) was obtained by averaging CRS incidence in the United States from 2001 to 2008 (most recent data) [8]. The number of live births was obtained from *National Vital Statistics Reports *[9,10]. Data on the number of live births per year (*γ *) were averaged over 2001-2009 (most recent data) and rounded to the nearest thousand. Average values were used for vaccine era CRS incidence and the number of live births because the annual values changed very little over the respective time periods of interest. Although vaccine era CRS incidence and live birth data were only available through 2008 and 2009, respectively, it was assumed that these data would remain relatively constant through 2010.

**Table 1:** Model parameters, estimates, and lower and upper limits

Parameter	Definition	Lower Limit	Estimate	Upper Limit
*α *	Vaccine era CRS incidence (cases per 10,000 live births)^a, b ^	-	0.00	-
*β *	Pre-vaccine era CRS incidence (cases per 10,000 live births)^c ^	2.0	4.0	15
*γ *	Live births per year (rounded to the nearest 1000)^b, d ^	-	4,150,000	-
*δ *	Percentage of CRS cases presenting with an ASD	3.0%	7.4%	10.0%

All data are from the United States unless otherwise specified in Methods.

^a ^Averaged over 2001-2008.

^b ^Parameter was not varied in sensitivity analyses (no lower and upper limit given).

^c ^Lower limit, upper limit, and estimate correspond to endemic, epidemic, and overall CRS incidence, respectively.

^d ^Averaged over 2001-2009.

Explicit data on pre-vaccine era CRS incidence in the US are limited. The estimate of CRS incidence (*β *) and lower and upper limits of this parameter were taken from a study by Stray-Pedersen [11], which modeled pre-vaccine era CRS incidence in Norway. In general, the values of pre-vaccine era CRS incidence reported by Stray-Pedersen are supported by research from other countries, although individual estimates of CRS incidence vary. Compared to the Norway study, CRS surveillance in Jamaica returned the same overall estimate of CRS incidence (4.0 cases per 10,000 births) [12]. A slightly higher estimate of approximately 5 CRS cases per 10,000 live births was reported from mathematical modeling of pre-vaccine era CRS incidence in Australia [13]. Mathematical modeling by Cutts and Vynnycky [14] yielded overall estimates of CRS incidence in the range of 17 cases per 10,000 live births in some developing regions. These estimates had very wide ranges [14], however, and other studies have only reported such high incidence rates during outbreaks [15]. Therefore, taking a conservative approach, we chose to use the value reported from Norway and Jamaica. The Norway and Jamaica studies also obtained the same estimate of endemic CRS incidence (2.0 cases per 10,000 live births) [11,12]. This value is similar to the estimated 0.81 to 1.27 CRS cases per 10,000 live births derived from a retrospective review of medical records in Morocco [16] and to the estimated 1 case per 10,000 live births obtained from active surveillance in Yangon, Myanmar [17]. In addition, a review by Cutts, et al. [15], summarized estimates of epidemic CRS incidence from several countries, ranging from 6 CRS cases per 10,000 live births in Trinidad and Tobago to 22 cases per 10,000 live births in Panama. Within this range fell the Stray-Pedersen estimate of 15 CRS cases per 10,000 live births [11], and we included this value in our calculations.

The percentage of CRS cases presenting with an ASD (*δ *) was obtained from the Chess study of 243 preschool children with CRS [7]. In the Chess study, eighteen children had either the "full" syndrome (meeting all diagnostic criteria) or "partial" syndrome (meeting some but not all diagnostic criteria) of autism, for a total prevalence of 7.4% [7]. Autism diagnosis was based on Kanner's original description of the disorder, published in 1943 [7]. According to Chess, Kanner's classical criteria for autism included "extreme autistic aloneness," language abnormalities, stereotypic relations to the environment, and a lack of affective human contact [7]. In our model, the value of 7.4% was set as the overall estimate of the percentage of CRS cases presenting with an ASD, and lower and upper limits were set at 3.0% and 10.0%, respectively. These limits are designed to be conservative, considering that 1) quantitative data on the CRS-ASD association are limited and 2) current ASD diagnostic criteria are broader and more inclusive than Kanner's classical criteria [18-20].

The estimates in Table 1 were used to calculate the numbers of CRS and ASD cases prevented by rubella vaccination from 2001 through 2010. A one-way sensitivity analysis of the number of CRS cases prevented was performed by varying pre-vaccine era CRS incidence. It is somewhat unreasonable to extend an "endemic" or "epidemic" estimate over a ten-year period because rubella epidemics typically occur every six to ten years in an unvaccinated population [21]. Nevertheless, we decided to employ the endemic estimate to represent the "most conservative scenario," in which CRS incidence remains low in spite of a lack of rubella vaccination. In contrast, extension of the epidemic estimate represents the "least conservative scenario," in which rubella transmission is sustained at high levels in an unvaccinated population. A two-way sensitivity analysis of the number of ASD cases prevented was performed by simultaneously varying pre-vaccine era CRS incidence and the percentage of CRS cases presenting with an ASD.

## Results

Using equations 1 and 2 and the parameter estimates given in Table 1 rubella vaccination prevented an estimated 16,600 CRS cases and 1228 CRS-associated ASD cases in the United States from 2001 through 2010. By changing pre-vaccine era CRS incidence from 2.0 cases per 10,000 live births (most conservative scenario) to 15 cases per 10,000 live births (least conservative scenario), the estimated number of CRS cases prevented ranged from 8300 to 62,250 (Table 2). Corresponding ASD prevention estimates ranged from 614 to 4607 cases (Table 3). Varying the percentage of CRS cases presenting with an ASD caused considerable, though less dramatic, changes in ASD prevention estimates. When the percentage of CRS cases presenting with an ASD was altered from the lower limit of 3.0% to the upper limit of 10.0%, an estimated 498 and 1660 ASD cases were prevented, respectively (Table 3).

**Table 2:** One-way sensitivity analysis of the number of CRS cases prevented by rubella vaccination, 2001-2010.

Pre-Vaccine Era CRS Incidence	Number of CRS Cases Prevented
Endemic (2.0 cases/10,000 live births)	8300
Overall (4.0 cases/10,000 live births)	16,600
Epidemic (15 cases/10,000 live births)	62,250

Pre-vaccine era CRS incidence was varied to give estimates of the number of CRS cases prevented under various rubella transmission scenarios.

**Table 3:** Two-way sensitivity analysis of the number of ASD cases prevented by rubella vaccination, 2001-2010.

Pre-Vaccine Era CRS Incidence	Percentage of CRS Cases Presenting with an ASD		
	
	Lower Limit	Estimate	Upper Limit
**Endemic**	249	614	830
**Overall**	498	1228	1660
**Epidemic**	1868	4607	6225

Pre-vaccine era CRS incidence and the percentage of CRS cases presenting with an ASD were varied simultaneously to give estimates of the numbers of CRS-associated ASD cases prevented.

Combining variations in pre-vaccine era CRS incidence and the percentage of CRS cases presenting with an ASD, an estimated minimum of 249 ASD cases and an estimated maximum of 6225 ASD cases were prevented by rubella vaccination from 2001 through 2010 (Table 3). Due to the expansion of ASD diagnostic criteria over time, using the upper limit of the percentage of CRS cases presenting with an ASD may more accurately reflect cases of ASD prevented using current diagnostic criteria. Employing the upper limit of the percentage of CRS cases presenting with an ASD, an estimated 1660 ASD cases (range: 830 to 6225 cases) were prevented by rubella vaccination from 2001 through 2010.

## Discussion

By preventing CRS, rubella vaccination has prevented hundreds, and perhaps thousands, of ASD cases from 2001 through 2010 in the US. These results demonstrate that the CRS-ASD association is not trivial, though the prevented cases represent only a small fraction of current ASD prevalence.

This study relies on estimates from Chess's evaluation of children with CRS. Fombonne, et al., published on the link between autism and other medical disorders in 1997 [22]. The investigators found the rate of CRS among autistic children to be 0.6%, but the association between autism and CRS was not significant. However, given the low overall prevalence of CRS in the population, the relatively small sample size (174 children with autism) would not have been sufficient to detect an association [22]. In addition, the controls selected by Fombonne and colleagues were children with other "medical and developmental problems" and "intellectual deficits," conditions which are also associated with CRS [1,2,22]. The findings of Fombonne and colleagues cannot be directly compared to Chess's estimates because Fombonne's study examined the relationship between CRS and autism following implementation of rubella vaccination.

According to Castillo-Solórzano, et al. [23], "In the United States the lifetime cost of treating a patient with CRS is estimated at more than $200,000." Using the CRS prevention estimates described in Results, rubella vaccination saved the US between $1.7 billion and $12.5 billion by preventing CRS from 2001 to 2010. These cost savings do not include the prevention of stress and emotional suffering for individuals with CRS and their families. Furthermore, since pre-vaccine era CRS incidence was based on the number of live births, these estimates do not account for stillbirths, miscarriages, or induced abortions related to CRS.

Our study is subject to a number of limitations. Because CRS and ASD prevention were estimated through mathematical modeling, the accuracy of prevention estimates is determined by the model parameters. Of the model parameters, estimates of vaccine era CRS incidence and the number of live births per year are based on recent data from reliable sources and, therefore, are expected to be accurate. Although data on these parameters were only available through 2008 and 2009, respectively, substantial changes in these parameters were not expected to occur before the end of the ten-year period (2001 to 2010).

Our results are limited in that we had to rely on the 1971 Chess study [7] for estimates of the percentage of CRS cases presenting with an ASD; no comparable data were available elsewhere. However, this limitation should lead to underestimation of ASD prevention, since ASD diagnostic criteria have expanded since the Chess study was conducted [20]. With the expansion of Kanner's classical criteria, we expect that more children with CRS would be diagnosed with an ASD today. Additionally, we used data from other countries to estimate pre-vaccine era CRS incidence in the United States because specific data for the US were not available.

Among parents who choose not to have their children vaccinated, vaccine safety concerns are often cited as the reason for vaccine refusal [24]. In a survey of pediatricians and family physicians in the United States in 2000, 69% of physicians perceived a "substantial increase" in parental concerns about vaccine safety [25]. Regarding MMR vaccination, a prospective cohort study of children in the United Kingdom found that 74% of parents of unvaccinated children made a "conscious decision" not to have their children vaccinated with the combined MMR vaccine [26]. Many of these parents claimed to be concerned about vaccine safety, including the potential association between the vaccine and autism. A smaller fraction cited "negative media attention" as a reason for their decision [26]. In the US, where monovalent rubella vaccine is not available, MMR vaccination is the only means of rubella vaccination.

Despite claims that MMR vaccination causes autism, research does not support this association [27-30]. These claims are also ironic in light of our results, which demonstrate that MMR vaccination (through the rubella component of the vaccine) actually prevents cases of autism and other ASDs through the prevention of CRS. Although a disparity between public opinion and scientific fact is evident, physicians and other healthcare providers are in a prime position to address the issue.

## Conclusions

Our findings highlight the importance of rubella vaccination in prevention of CRS and resultant autism spectrum disorders. These findings should be incorporated into parental communication materials. Clinicians and public health professionals should continue to educate patients and the public about the benefits of rubella vaccination.

## Abbreviations

CRS: congenital rubella syndrome; ASD: autism spectrum disorder; MMR: measles-mumps-rubella;

## Competing interests

The authors declare that they have no competing interests.

## Authors' contributions

All authors participated in designing the study and drafting the manuscript. BEB conducted the study with guidance and coordination from SBO. AMNB and SBO conceived of the study. All authors read and approved the final manuscript.

## Pre-publication history

The pre-publication history for this paper can be accessed here:

http://www.biomedcentral.com/1471-2458/11/340/prepub
